# High glucose provokes microvesicles generation from glomerular podocytes via NOX4/ROS pathway

**DOI:** 10.1042/BSR20192554

**Published:** 2019-11-19

**Authors:** Mingzhen Li, Tian Zhang, Xin Wu, Yulin Chen, Lirong Sun

**Affiliations:** NHC Key Laboratory of Hormones and Development (Tianjin Medical University), Tianjin Key Laboratory of Metabolic Diseases, Tianjin Medical University Chu Hsien-I Memorial Hospital and Tianjin Institute of Endocrinology, Tianjin 300134, China

**Keywords:** high glucose, microvesicles, NOX4, podocyte

## Abstract

Microvesicles (MVs) were involved in the pathogenesis of many diseases, such as cardiovascular diseases and diabetes. Oxidative stress played a key role in the development and progression of diabetic nephropathy (DN). Our aim of the present study was to investigate whether high glucose (HG) could provoke MVs generation from podocytes and its potential mechanism. Mouse podocyte clone 5 (MPC-5) was stimulated by HG. The intracellular reactive oxygen species (ROS) of podocytes were measured by fluorescence microscopy with the probe of CM-H_2_DCFDA and MitoSOX™. Antioxidants N-Acetyl-l-cysteine (NAC) and α lipoic acid (α-LA) were used to treat podocytes after HG stimulation. The rate of podocyte apoptosis was evaluated with Annexin V-FITC by flow cytometry. NOX4 expression was examined and siRNA were performed to explore the mechanism of MVs generation. The quantities of MVs from MPC-5 cells was significantly increased (*P*<0.05) by 4.6-times after 30 mM glucose stimulation, accompanied with double increased apoptosis. Cellular ROS generation was increased by HG at the peak of 48 h stimulation. HG-induced MVs were significantly decreased by 52.9% after pretreatment by antioxidant NAC. Nevertheless, mitochondrial ROS in podocytes reached a peak at 4 h stimulation, but specific antioxidant α-LA had no effect on the production of MVs (*P*>0.05). Levels of NOX4 mRNA and protein expression were significantly up-regulated by HG (*P*<0.05). Podocyte-derived MVs by HG were eliminated by NOX4 siRNA. HG can provoke MVs generation from glomerular podocytes through ROS/NOX4 pathway, not from mitochondrial pathway.

## Introduction

Diabetic patients also show hypercoagulability and platelet hyperaggregability, with increased levels of platelet activation markers such as P-selectin, soluble CD40 ligand, and microvesicles (MVs) [1,2]. It was shown that MVs shedding from cell membranes, were involved in the pathogenesis of many diseases by the transfer of bioactive molecules and modification of extracellular milieu and recipient cells [3]. MVs may participate in hemostatic and inflammatory responses, neovascularization, cell survival, and apoptosis, processes which are involved in atherothrombosis [4]. Recently, Burger et al. [5] assessed the effects of high glucose (HG) on endothelial MV (eMV) formation, composition, and signaling in cultured human umbilical vein endothelial cells (HUVECs), indicating that elevated glucose is a potent stimulus for eMV formation, which also alters their molecular composition leading to increased bioactivity. Such effects may contribute to progressive endothelial injury and subsequent cardiovascular complications in diabetes [5]. Thus, MVs may be associated with a higher incidence of vascular disease in diabetic patients through a mechanism that is unclear.

Diabetic nephropathy (DN) is the leading cause of chronic kidney failure. Approximately 30–40% of patients with type 1 or type 2 diabetes develop evidence of nephropathy [6,7]. Among the characteristic findings of DN, podocytes are involved in the development of glomerular hypertrophy, podocytopenia, glomerulosclerosis, and foot process effacement [8]. Together with glomerular endothelial cells and glomerular basement membrane, podocytes form the glomerular filtration barrier in the kidney. Podocyturia may be an expression of glomerular disease and is evaluated using urinary podocyte-specific molecules. Given their dysregulation in kidney disease, podocytes and their specific proteins and mRNA pose as attractive candidates as either diagnostic or predictor biomarkers of disease [9].

MVs might be associated with podocytes in diabetic patients, however, the related mechanism was still unclear. Moreover, whether MVs played a role in the pathogenesis of DN was also unknown. In the present study, we investigated whether HG could provoke the generation of MVs, the related mechanisms were also evaluated.

## Materials and methods

### Cell culture

Conditional immortalized mouse podoyte clone 5 (MPC-5) was kindly and generously provided by Professor Peter Mundel (Mount Sinai Medical College, U.S.A.) and Professor Ding Jie (First Hospital of Peking University). MPC-5 was proliferated at 33r and 5% CO_2_ with RPMI 1640 containing IFN-γ (10 IU/ml), and differentiated at 37t and 5% CO_2_ with RPMI 1640.

### Reagents and chemicals

The Zymuphen MP-Activity ELISA kit was purchased from HYPHEN BIOMED, U.S.A. Antioxidants N-Acetyl-l-cysteine (NAC) and α lipoic acid (α-LA) were purchased from Sigma, U.S.A. The probe of CM-H_2_DCFDA and MitoSOX™ Red were purchased from Molecular Probes, U.S.A. Annexin V-FITC was purchased from Tianjin Sungene Biotech Co. Ltd. Antibodies against NOX4 was purchased from Cell Signaling Technology, U.S.A. PCR primers were synthesized by Takara.

### MVs detection

The Zymuphen MP-Activity ELISA kit was used to assay the concentration of MVs according to manufacturer’s protocol. Briefly, conditional cellular medium was washed twice with phosphate buffer solution (PBS) and centrifuged at 2000 rpm for 10 min, followed by centrifugation of supernatant at 100000×***g*** for 90 min. The final precipitation was suspended with PBS and ready for MVs measurement following the protocol.

### The effect of antioxidants on MVs generation

Antioxidants, including NAC or α-LA were administrated to MPC-5 one hour before HG stimulation.

### Apoptosis of podocytes

For apoptosis analysis, podocytes were divided into three groups: normal control, hyperosmotic control (mannitol 30 mM) and HG (30 mM). Annexin V-FITC/PI apoptosis kit (Sungene Biotech, Tianjin, China) was used to detect the apoptosis of podocytes according to manufacturer’s instructions. After 24-h stimulation, cells were trypsin-digested and collected by centrifugation at 1000 rpm for 10 min. Cells were incubated by Annexin V-FITC or PI at room temperature for 10 min, and then were ready for flow cytometry.

### Reactive oxygen species detection

MPC-5 cells were stimulated by HG (30 mM) for given time. Intracellular reactive oxygen species (ROS) was detected by the probe CM-H_2_DCFDA, which can be converted in to highly fluorescent DCF-DA upon oxidation by ROS in the living cells, and assessed by fluorescent microscope (Olympus, Japan). Fluorescent intensity was quantified by Image-Pro Plus 6.0 software. MitoSOX™ Red is live-cell permeant and is rapidly and selectively targeted to the mitochondria. Once the indictor is oxidized by superoxide (major ROS source from mitochondria) and binds to nucleic acids, the reagent exhibits red fluorescence, followed by the assessment by flow cytometry (BD Calibur, U.S.A.).

### Quantitative real-time PCR

Total RNA from MPC-5 was isolated with TRIzol Reagent (Invitrogen, Carlsbad, CA) and converted into cDNA (TAKARA, Dalian, China). Real-time fluorescence quantitative PCR was applied to measure the expression of Nox4 mRNA, using the primers (TAKARA, Dalian, China) as following: Nox4: sense 5′-CgATTCCgggATTTgCTACTg-3′, antisense 5′-CCTCAAATgggCTTCCAAATg-3′; GAPDH: sense 5′-CAAggTCATCCATgACAACTTTg-3′, antisense 5′-gTCCACCACCCTgTTgCTgTAg-3′. PCR amplification was performed for 39 cycles as following: the initial denaturation at 95°C for 30 s, annealing at 60°C for 30 s, at 95°C for 10 s with final extension at 65°C for 5 s. 2^−ΔΔ*C*_t_^ was used to calculate the relative expression of Nox4 mRNA.

### Nox4 protein measured by Western blot

Total protein from MPC-5 was extracted with RIPA lysis buffer. After HG stimulation for different time points, cell lysis samples were subjected to SDS/PAGE electrophoresis, followed by transfer to PVDF membranes, and blockage for 2 h at room temperature with 5% skim milk. The primary antibody rabbit polyclonal anti-NOX 4 (1:2000) or Mouse monoclonal anti-β-Tubulin (1:5000) was incubated overnight at 4°C on the shaker. The membranes were incubated with horseradish peroxidase–conjugated goat anti-rabbit or anti-mouse antibody (Sungene Biotech, Tianjin, China). The specific proteins were detected using an enhanced chemiluminescence (ECL) Western blotting kit (Advansta, San Jose, CA). ImageJ was applied for targeted bands analysis.

### Nox4 siRNA and cell transfection

Experiments were performed with selected specific small interfering RNA sequence as follows (siRNAs; GenePharma, Shanghai, China), Nox4: sense 5′-CCAGUGGUUUGCAGAUUUATT-3′, antisense 5′-UAAAUCUGCAAACCACUGGTT-3′; negative-siRNA: sense 5′-UUCUCCGAACGUGUCACGUTT-3′, antisense 5′-ACGUGACACGUUCGGAGAATT-3; FAM-siRNA: sense 5′-UUCUCCGAACGUGUCACGUTT-3′, antisense 5′-ACGUGACACGUUCGGAGAATT-3′ as positive control. siRNA conditions were optimized. NOX 4 siRNA was transfected by the Lipofectamine 2000 transfection reagent (Invitrogen, U.S.A.) according to the manufacturer’s protocol. Cells were used for experiments at 48 h after transfection.

Cells were randomly grouped into the following four groups: (1) without NOX 4 siRNA and HG (Glu-, NOX4 siRNA-); (2) with NOX 4 siRNA but without HG (Glu-, NOX4 siRNA+); (3) with HG, but without NOX4 siRNA (Glu+, NOX4 siRNA-); (4) with HG and NOX4 siRNA (Glu+, NOX4 siRNA+).

### Statistical analysis

All the data were analyzed by SPSS 18.0. Normally distributed data are shown as means ± SEM. Comparisons among three or more groups were performed by one-way analysis of variance (ANOVA), followed by the Student–Newman–Keuls (SNK) test. Comparisons between two groups were analyzed using the Student’s unpaired, two-tailed *t* test. Comparisons between two groups of non-normally distributed data used Games–Howell test. *P*<0.05 was considered as statistically significant.

## Results

### Quantities of MVs

**Figure 1:**
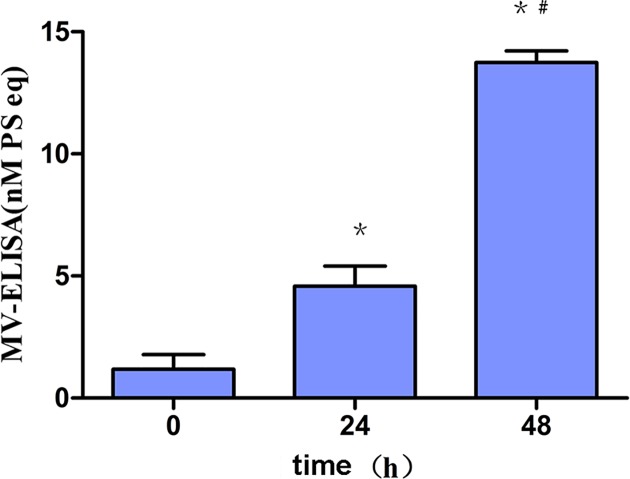
HG-induced MVs generation from podocytes MPC-5 podocytes were randomly exposed to HG for 0, 24 and 48 h. MVs concentration was remarkably and significantly elevated (4-folds increase for 24 h, and 12-folds increase for 48 h, *P*<0.001). *, *P*<0.001, compared with control (0 h); ^#^, *P*<0.001, compared with 24 h.

HG significantly provoked total MVs generation from MCP-5 in a time-dependent manner. MVs (1.18 ± 0.49 nM) were four-folds increased at 24 h (4.59 ± 0.67 nM), and 12-folds increased at 48 h (13.75 ± 0.39 nM) after stimulation, compared with the control (*P*<0.001) (Figure 1).

### Apoptosis of podocytes after HG stimulation

**Figure 2:**
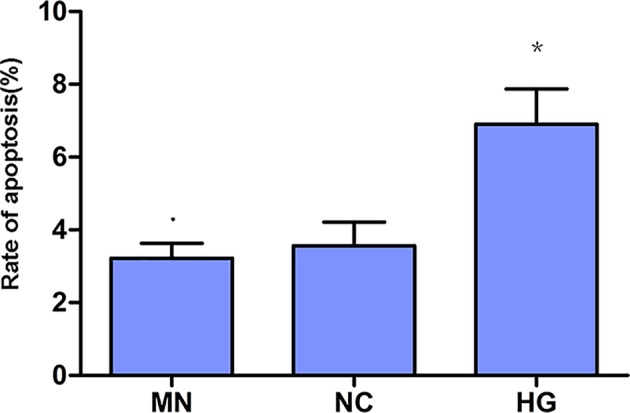
HG-induced apoptosis of podocytes Podocytes were exposed to HG for 24 h, with manitol as hyperosmotic control. Apoptosis of podocytes was increased significantly two times as control (*P*<0.01). There was no significant difference of apoptosis between mannitol and control groups. *, *P*<0.01, compared with NC.

After HG stimulation, apoptosis of podocytes was detected by flow cytometry (Figure 2). Compared with NC, HG significantly increased the apoptosis of podocytes by almost one-fold (*P*<0.01). Mannitol had no effect on the apoptosis of MPC-5 (*P*>0.05).

### ROS generation after HG stimulation

**Figure 3:**
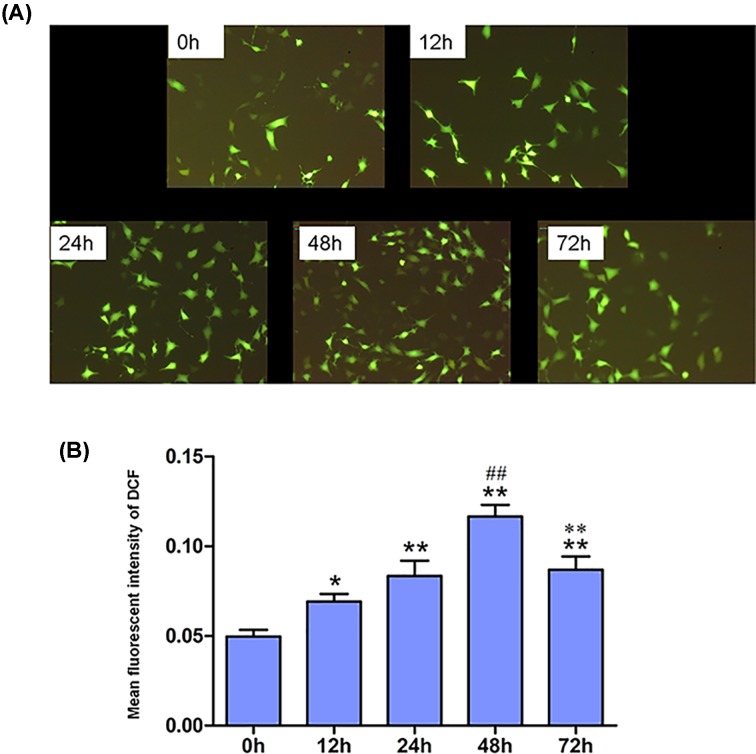
Time course of intracellular ROS detection (**A**) Representative microphotographs of fluorescent staining showing CM-H_2_DCFDA (green) (×100). Scale bar = 50 μm. (**B**) Fluorescent intensity of intracellular ROS (*n*=4). *, *P*<0.01, compared with 0 h; **, *P*<0.001, compared with 0 h; ^##^, *P*<0.001, compared with 24 h; ***, *P*<0.001, compared with 48 h.

Cellular ROS generation was increased in a time-dependent manner after HG stimulation (Figure 3A). ROS increased over time, and peaked at 48 h after stimulation by HG (1.4-fold at 12 h, 1.7-fold at 24 h, 2.3-fold at 48 h and 1.7-fold at 72 h after stimulation, respectively, *P*<0.01) (Figure 3B).

**Figure 4:**
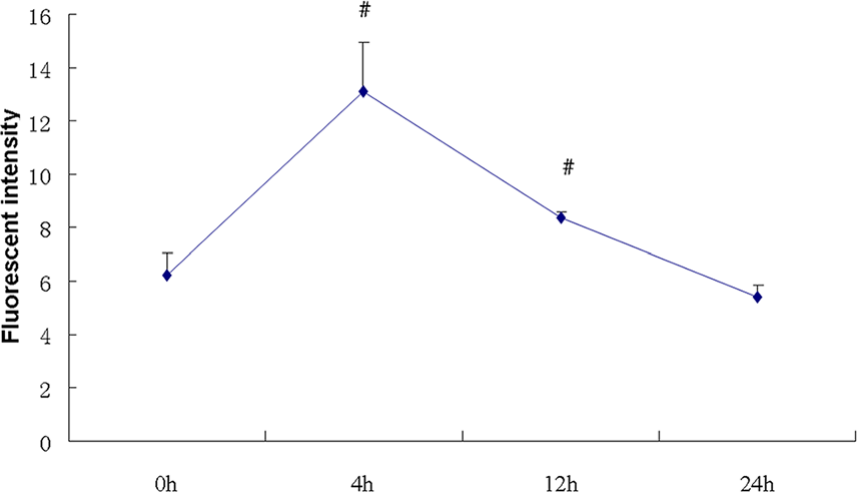
Time course of mitochondrial ROS detection Mitochondrial ROS was detected by flow cytometry at given time point. ROS was increased at 4 h, and also reached peak, and then fell to baseline at 24 h (*n*=4). ^#^, *P*<0.05, compared with 0 h.

Mitochondrial ROS was increased over time, detected by flow cytometry with the probe of mitoSOX. Fluorescent intensity of mitoSOX reached peak by double increase at 4 h (*P*<0.05), and decreased back to baseline at 24 h (Figure 4).

### Effect of antioxidants on the generation of MVs

**Figure 5:**
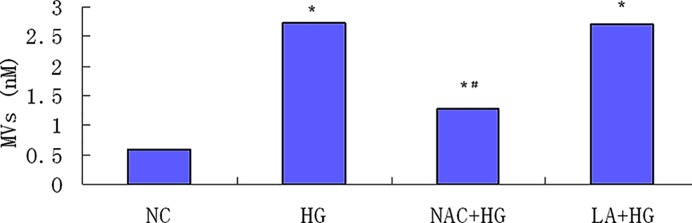
Antioxidants effect on HG-induced MVs generation Podocytes were randomly treated with NAC or LA 1 h before exposure to HG for 24 h. NAC significantly decreased HG-induced MVs generation by 52.9% (*P*<0.05), but not LA (*P*>0.05) (*n*=4). *, *P*<0.05, compared with NC; ^#^, *P*<0.05, compared with HG.

Antioxidants were pretreated 1 h before HG stimulation. MVs were decreased significantly by 52.9% with the administration of NAC (1.29 ± 0.12 nM), compared with HG group (2.74 ± 0.11 nM) (*P*<0.05), but it was still higher than NC (0.64 ± 0.11 nM). The effect of α-LA on generation of HG-induced MVs was not observed (2.73 ± 0.12 nM) (*P*>0.05) (Figure 5).

### NOX4 expression after HG exposure

**Figure 6:**
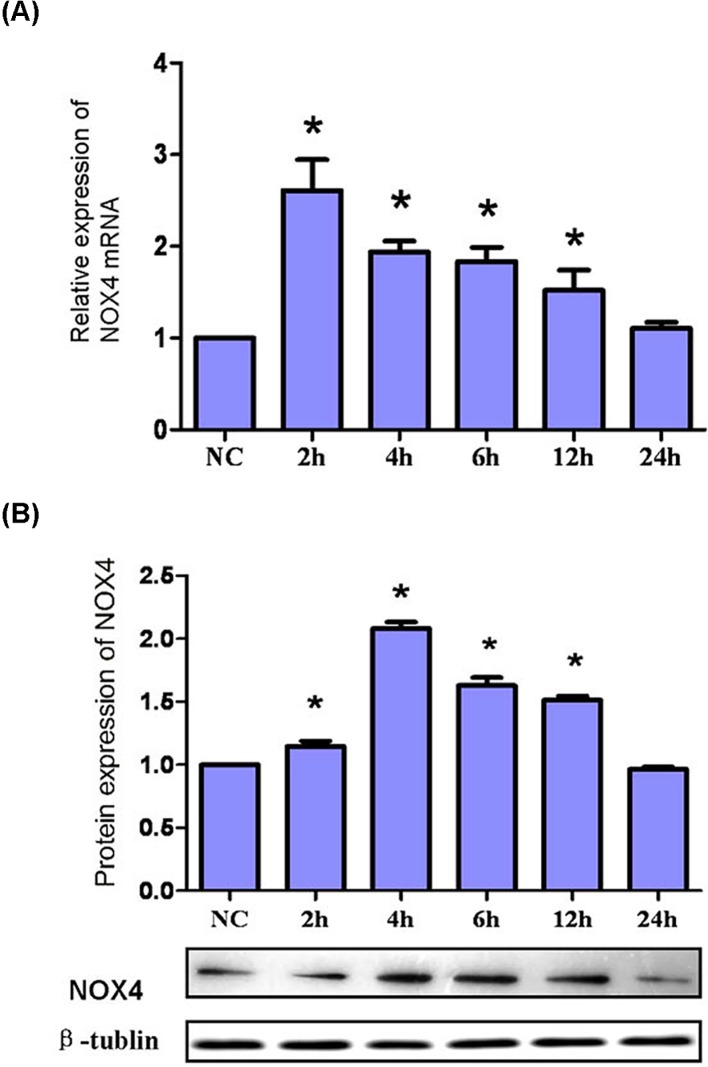
mRNA expression and protein expression of NOX4 (**A**) Time course of NOX4 mRNA expression. Podocytes were randomly exposed to HG for given time point. NOX4 mRNA expression was up-regulated immediately at 2 h to 2.6-folds of NC, and then fall back gradually to the baseline at 24 h. *, *P*<0.001, compared with NC (*n*=4). (**B**) Time course of NOX4 protein expression. Podocytes were randomly exposed to HG for given time point. Representative Western blot was shown, and β-tubulin was used as a loading control. NOX4 protein expression was up-regulated immediately from 2 h, and peaked at 4 h to 2.08-folds of NC, and then fall back gradually to the baseline at 24 h. *, *P*<0.01, compared with NC (*n*=4).

NOX4 mRNA expression began to increase and reached the peak after HG exposure for 2 h (*P*<0.001), then it decreased to baseline at 24 h (Figure 6A). NOX4 protein expression showed same pattern after HG, but reached peak at the time point 4 h (*P*<0.001) (Figure 6B).

### MVs generation after NOX4 RNA silence

**Figure 7:**
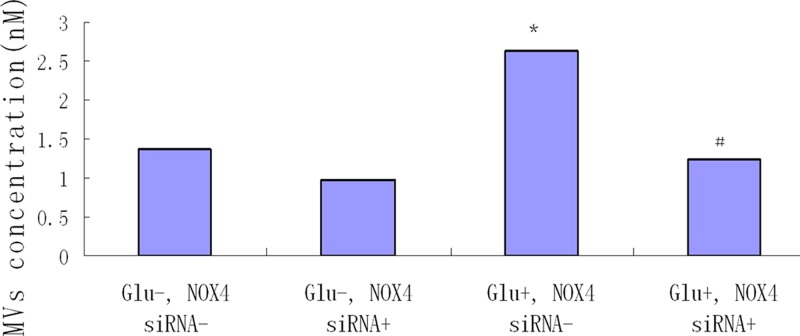
Effect of silencing NOX4 RNA on HG-induced MVs generation in podocytes Podocytes were transfected with NOX4 siRNA (125 nM), and treated with or without HG for 24 h. *, *P*<0.01, compared with groups 1 and 2. MVs from group 4 (Glu+, NOX4 siRNA+) were comprehensively and significantly blocked, compared with group 3 (*P*<0.01). ^#^, *P*<0.01, compared with group 3 (*n*=4).

MVs from group 3 (Glu+, NOX4 siRNA-) (2.63 ± 0.22 nM) were significantly increased compared with groups 1 and 2 and it was almost two times compared with control (*P*<0.01). After silencing NOX4 RNA, MVs from podocytes in group 4 (Glu+, NOX4 siRNA+) (1.25 ± 0.05 nM) were comprehensively and significantly inhibited compared with group 3 (*P*<0.01), Moreover, there was no significant difference of the MVs from podocytes between group 4 (1.25 ± 0.05 nM) and group 1 (1.38 ± 0.07 nM) after siRNA treatment (*P*>0.05) (Figure 7).

## Discussion

MVs, sizing from 100 nm to 1 µm, are shed by the plasma membrane of living cells [10], through a mechanism supported by cytoskeletal remodeling. MVs are dramatically increased in pathological conditions, such as inflammation, oncogenic transformation, or other strong cellular stresses [11–16]. Recent researches illustrated that MVs could transfer bioactive molecules, including proteins, DNA, mRNA, and miRNA, and might play an important role in future diagnostic and therapeutic strategies [11]. Several miRNAs (such as miR-192, 210, 29 family) in patients’ blood or urinary sediments were highlighted in DN [17–19]. Here, we are the first to use MPC-5 (the immortal podocyte cell model) to investigate the generation of MVs from podocytes exposed to HG, and its potential mechanisms. Our findings showed that MVs generation from podocytes was dramatically increased after the exposure to HG, which was accompanied with increased apoptosis. Intracellular total ROS was increased by HG stimulation in a time-dependent manner. Both increased ROS and MVs were inhibited by the antioxidant NAC. This outcome implied that MVs were strongly correlated with apoptosis and were consistent with previous studies showing that MVs can either promote or protect against cellular apoptosis, depending on the stimulator and cell line from which the MVs were generated [20–22].

HG induced generation of MVs from MPC-5 cells and oxidative stress could be detected. This finding could help increase the understanding of why diabetics are more thrombogenic than non-diabetics. MVs are typically generated after cell activation or apoptosis following the disturbance of membrane phospholipid asymmetry arising from the altered activity of pumps that are involved in phospholipid transport [23].

Oxidative stress has been recognized as one of the central pathogenesis of podocyte injury in DN [24]. ROS production is considered to be significantly increased in podocyte in DN, however, whether ROS source is predominantly from NADPH oxidase and mitochondria in diabetes is still controversial [25,26]. In the present study, we used two probes to detect ROS from podocytes exposed to HG, one is CM-H_2_DCFDA, for intracellular total ROS and the other is MitoSOX™, especially for mitochondrial ROS. Our results illustrated that both intracellular total ROS and mitochondrial ROS were significantly increased by HG in a time-dependent manner. In order to explore the relationship between ROS and MVs production, we also administrated podocytes with two antioxidants including NAC and mitochondria-targeted α-LA. We found that NAC significantly inhibited HG-induced MVs production by 52.9% from podocytes. Although LA is an essential cofactor for energy production in the mitochondria, it also acts as a powerful antioxidant and a free radical scavenger, due to the thiol bond [27,28]. However, we did not observe the effect of α-LA on podocyte-derived MVs. Therefore, ROS from mitochondria might not be the culprit for the MVs generation.

Based on our results, we demonstrated that NADPH oxidase might exert an important role in podocyte-derived MVs by HG. NOX-4, as a member of the NADPH oxidase family, has been considered as the key oxidase in increased ROS production in podocyte in DN [29]. We examined NOX-4 expression by quantitative real-time PCR and Western blot. Our findings showed that the expressions of mRNA and protein for NOX-4 were increased significantly by HG in a time-dependent manner, which was consistent with previous studies [30,31]. After we silenced NOX-4 RNA, HG-induced MVs production in podocytes was blocked comprehensively. Our findings indicated that NOX-4 was crucial for HG-induced MVs.

In conclusion, since podocyte MVs are complex and heterogeneous under different conditions, they might be a novel useful biomarker for DN [32], or have cross-talk with other adjacent cells in the kidney through autocrine or paracrine signaling pathway [33,34]. More evidences will be needed for the elaboration of podocyte MVs.

Our study revealed that HG could provoke MVs generation from podocytes through NOX-4/ROS pathway. The potential roles of podocyte-derived MVs in DN need further study in future.

## Abbreviations

DN: diabetic nephropathy
eMV: endothelial microvesicle
HG: high glucose
IFN: interferon
MPC-5: mouse podocyte clone 5
MV: microvesicle
NAC: N-Acetyl-l-cysteine
NOX: nicotinamide adenine dinucleotide phosphate oxidase
PBS: phosphate buffer solution
ROS: reactive oxygen species
α-LA: α lipoic acid
